# Morphological Evolution of Physical Robots through Model-Free Phenotype Development

**DOI:** 10.1371/journal.pone.0128444

**Published:** 2015-06-19

**Authors:** Luzius Brodbeck, Simon Hauser, Fumiya Iida

**Affiliations:** 1 Institute of Robotics and Intelligent Systems, Department of Mechanical and Process Engineering, ETH Zürich, 8092 Zürich, Switzerland; 2 Department of Engineering, University of Cambridge, Cambridge CB2 1PZ, United Kingdom; 3 Biorobotics Laboratory, EPFL—Ecole Polytechnique Fédérale de Lausanne, 1015 Lausanne, Switzerland; University of Vermont, UNITED STATES

## Abstract

Artificial evolution of physical systems is a stochastic optimization method in which physical machines are iteratively adapted to a target function. The key for a meaningful design optimization is the capability to build variations of physical machines through the course of the evolutionary process. The optimization in turn no longer relies on complex physics models that are prone to the reality gap, a mismatch between simulated and real-world behavior. We report model-free development and evaluation of phenotypes in the artificial evolution of physical systems, in which a mother robot autonomously designs and assembles locomotion agents. The locomotion agents are automatically placed in the testing environment and their locomotion behavior is analyzed in the real world. This feedback is used for the design of the next iteration. Through experiments with a total of 500 autonomously built locomotion agents, this article shows diversification of morphology and behavior of physical robots for the improvement of functionality with limited resources.

## Introduction

The adaptation of physical shapes and structures is a fundamental mechanism which allows biological systems to survive in a large variety of environments. Through evolutionary adaptation some animals changed their morphologies to live on land instead of under water, and phenotypic plasticity allows plants to adapt their structures for the survival on an ontogenetic time-scale [1]. Today’s machines, in contrast, are highly restricted to their initial morphological configurations, and it is still a question whether machines can achieve a similar level of adaptability by adjusting their morphologies [2, 3, 4].

Inspired by nature, an engineered counterpart of evolutionary adaptation was investigated in the past [5, 6, 7]. The co-optimization of body and mind was demonstrated with the simulated evolution of virtual animal-like creatures [8, 9, 10, 11], which exploited evolutionary dynamics to generate variations of mechanical bodies as well as motor control circuitry for meaningful dynamic behaviors such as walking, running and swimming.

Artificial evolution was also brought to real-world systems. In a number of applications the solutions to engineering problems were evolved, such as the optimized shape for a satellite antenna [12], and it has been shown that electronic circuits can be optimized online for a specific task using evolutionary processes to adapt the configuration of the hardware [13, 14]. These results show that evolution can design unexpected solutions. However, there is only a limited number of mechatronic systems that can physically adapt their morphologies [15, 16, 17, 18]. Therefore, these systems are typically optimized in simulation, and only afterwards the results are reproduced in reality [19, 20]. Simulations allow to test thousands or millions of solutions in relatively short time, but they rely on the accuracy of the physics models employed that is often limited for many interaction types. The insufficient accuracy can lead to an inferior performance of the solution when tested in the real world, which is usually referred to as the “reality gap” [21, 22]. Another critical issue is the fact that developmental processes of physical systems are not considered in the simulated evolution [23], and it was not clarified how artificial evolution would be able to design “buildable” physical machines [24]. In their work on evolved Lego structures, Funes and Pollack constrain the simulation such that only structures which can be assembled evolve [25]. Kuehn and Rieffel on the other hand ensure the buildability of the passive structures they evolve by directly encoding executable fabrication instructions [26].

Here, we propose a model-free implementation for the artificial evolution of physical systems, to stochastically optimize the design of real-world machines. Machines are physically constructed and their performance is analyzed without simulation and human intervention to incrementally improve their functionality. While this model-free approach allows to avoid the reality gap and the selection of infeasible solutions, at least two problems need to be solved. First, given the limited resources in the real world, particularly time, an efficient improvement of functionality must be achieved in a limited number of evolutionary iterations. And second, physical constraints in the automatic construction of variations of machines have to be addressed because conventional automation technologies are typically designed for mass production rather than mass customization, thus the autonomous development of a large morphological diversity still remains to be a considerable challenge.

## Materials and Methods

### Process Overview

**Fig 1:**
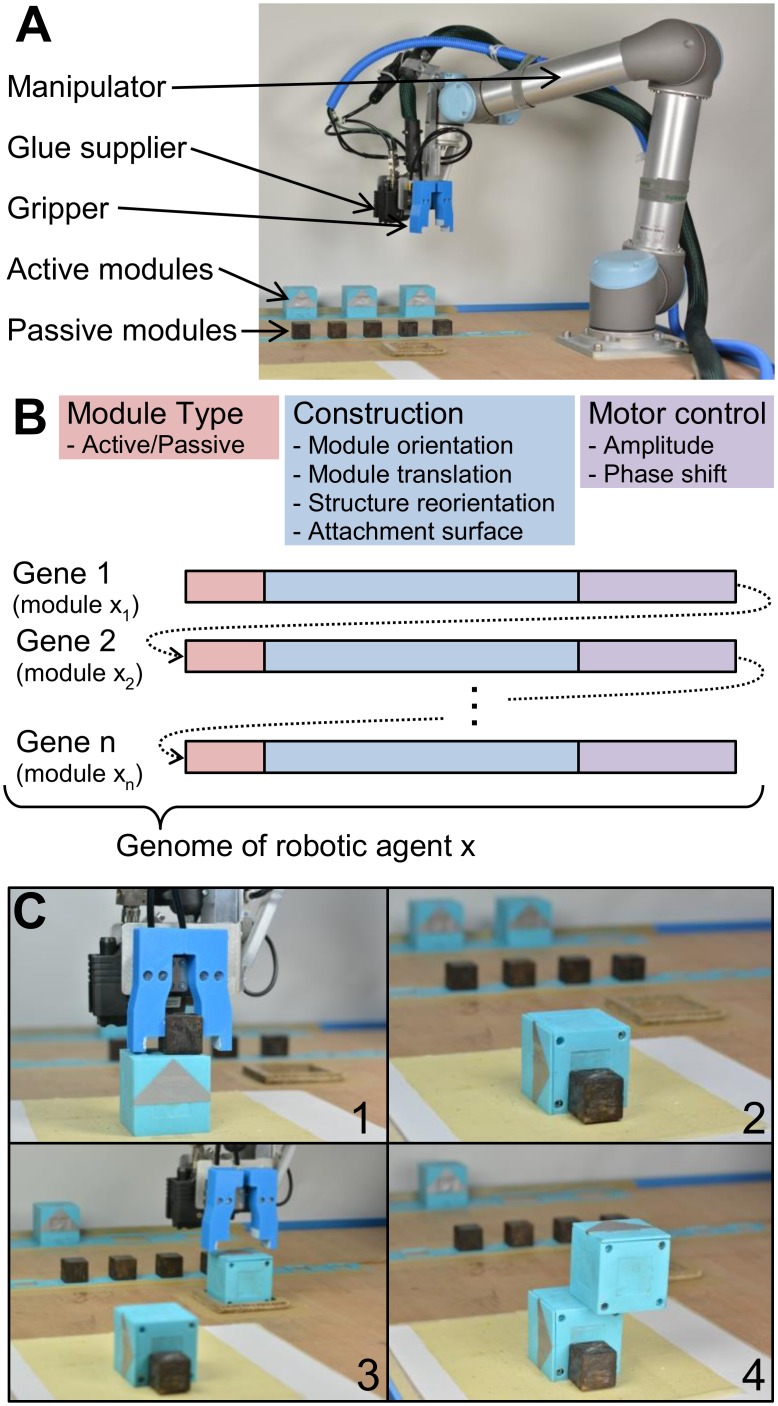
Developmental process. A “mother robot” (A) is used for the automatic assembly of candidate agents from active and passive modules. For the construction process, the robotic manipulator is equipped with a gripper and a glue supplier. Each agent is represented by the information stored in its genome (B). It contains one gene per module, and each gene contains information about the module types, construction parameters and motor control of the agent. A construction sequence encoded by one gene is shown in (C). First, the part of the robot which was encoded by the previous genes is rotated (C1 to C2). Second, the new module (here active) is picked from stock, rotated (C3), and eventually attached on top of the agent (C4).

The main enabling technology for the model-free artificial evolution is the implementation of an automated construction process for the stochastic optimization of mechanical designs in the real world [26]. In this framework a “mother robot” is considered, i.e. a robotic manipulator that assembles candidate agents from given resources (Fig 1A). Each of the constructed candidate agents is assembled from a set of passive and active cubic modules that the mother robot can easily manipulate. The mother robot performs a series of handling operations such as pick-and-place or rotation to rearrange the components and physically connect them with hot melt adhesive (HMA).

The evolutionary optimization in this framework is induced by the encoding of the physical construction processes in each agent’s genome. As shown in Fig 1B, our framework considers every candidate agent to be represented by a genome containing the information about the types of modules, construction parameters, and motor commands for active modules. The construction was constrained in a way that, on the one hand, the mother robot is capable to execute the relevant operations, and on the other hand, the outcome becomes sufficiently diverse. Given a genome as described here, the mother robot can reproduce a candidate agent, adding the modules one by one to the structure which eventually forms the candidate agent’s phenotype after completion of the operations (Fig 1B and 1C).

After the construction of each agent, the mother robot evaluates the phenotype according to a given fitness criterion, here the distance travelled from the initial position divided by the testing time. For the evaluation, the mother robot transports the candidate agent to a testing arena, where the agent’s motor modules are activated through wireless communication. The agent's behavior is recorded by an overhead camera and analyzed for the fitness evaluation (S2 Movie). The motor command parameters of active modules are evolved as part of the agent's genome, thus the evolutionary process can coevolve the agent’s “body” and “mind”. Once the fitness evaluation is finished, the agents are manually disassembled and remaining HMA is removed from the modules. The development and evaluation is repeated for all candidate agents in a generation, followed by the regular stochastic optimization employing a mixture of elitism as well as one-point crossover and mutation strategies (for details of the evolutionary process see the Algorithm section and S1 File). With the stochastic modification of the genomes, design variations of agents can emerge and eventually improve the functionality over generations.

### Hardware

The robotic manipulator (Universal Robots, UR5) is shown in Fig 1A. It has 6 degrees of freedom and in order to manipulate and connect the modules, the robot is equipped with two tools: A gripper and an HMA-supplier.

The gripper is a pneumatic two-fingered parallel gripper. The fingers are 3D-printed from acrylonitrile butadiene styrene (ABS). The fingers are designed such that objects of two different sizes can be gripped. For the connection of modules, the end-effector is equipped with an HMA supplier. The HMA material (ALFA Klebstoffe AG, ALFA H 5500/30) is automatically extruded from the jetting head (Robatech AG, SX 1N/296 Diamond) at a temperature of 165°C. The HMA material is supplied from a melting tank (Robatech AG, Concept B12) through a heated hose.

The active elements are used for the actuation of the locomotion agents and are of cubic shape with a side length of 6 cm and a total mass of 160 g (S1 Fig). A servo motor (Modelcraft, RS-3 JR) can rotate one of the cube’s surfaces. The active elements include a communication element (Sparkfun, Bluetooth Mate Silver), a microcontroller (Arduino, Pro Mini) to control the servo motor and a battery (Conrad Electronic AG, Conrad energy LiPo Akku 7.4 V, 800 mAh) to supply power. The case is 3D-printed from polylactic acid (PLA). The servo motors are controlled to oscillate with two control parameters: Amplitude and phase shift. At rest, the rotating plane is aligned with the body of the module. The control signal for the servo is switched between apexes, therefore speed of rotation is given by the hardware and the frequency is coupled to the amplitude. The amplitude can be set to five discrete values: 0°, 10°, 20°, 30°, and 40°, and the phase shift can be chosen among eight discrete values: 0°, 45°, 90°, 135°, 180°, 225°, 270° and 315°. To ensure that the rotation always starts from rest (initial aligned configuration), depending on the phase shift, a constant offset angle is added. As the passive element, wooden cubes with a side length of 3 cm and a mass of 30 g are used. The color of the passive cubes was changed to black for an easier detection in the image analysis.

### Control and Communication

The main controller is programmed in MATLAB and running on a desktop PC. The genome is fed into a MATLAB function which creates a virtual model of the agent—which is only used for verification—and simultaneously generates the robot commands for the manipulator.

The main controller communicates with the controller of the robotic manipulator via TCP/IP and with the active modules through a Bluetooth connection. The robotic manipulator runs its own controller, which also controls the gripper and the HMA supplier of the end-effector. During the building process, the main controller executes the control sequence and sends the commands with parameters to the manipulator controller, which runs them sequentially in a feed-forward manner.

In the testing phase, the commands for the active modules are sent by the main controller via Bluetooth to the modules. The onboard microcontroller interprets the command string and starts the oscillatory movement of the servomotor with the transmitted amplitude and phase shift.

### Construction and Testing Environment

For the ease of assembly, the construction workspace is structured into the following three parts: storage grid, centering frame, and construction surface. The storage grid allows for the manipulator to access the elements (active and passive) at predefined locations. When rotating active elements, they are placed in the centering frame after every rotation to avoid that position errors sum up during the manipulation process. The centering frame is a cardboard frame with a foil lip at its inner edge which acts as a spring to push misaligned elements towards the frame’s center. The construction surface is covered with a soft, slightly sticky ground to ensure good ground contact and error tolerance (3 mm foam rubber covered with masking tape, sticky side up).

The locomotion tasks were done on three different grounds. The first is a hard flat surface (plywood covered with white fabric). The second ground is a carpet (thickness 1 cm) and for the third environment a 1 cm layer of polyurethane foam was added on top of the carpet.

### Algorithm

The optimization is based on an evolutionary algorithm, which iteratively improves the construction sequence of locomotion agents. An ordered set of one to five genes contains the complete information describing an individual, the genome. The algorithm starts initializing the genomes of the first generation of candidate agents. Evolution then is performed through mutation, where components in one gene are modified or single genes are added or deleted, and crossover, where a new genome is formed by merging genes from two individuals. The genome then is interpreted and expressed into the phenotype which describes the appearance of the final agent.

The design of the encoding is based on a trade-off. Any genome generated with the chosen encoding should be buildable, which demands simplicity. On the other hand it has to be powerful enough to generate complex agents. To enable a direct transfer of the encoded information to the building process, the building sequence is directly encoded, i.e. each genome specifies a series of operations to be executed in the building process [26].

An operation consists of several elementary actions, e.g. open gripper or move to another position. A gene defines a sequential series of operations, executed in a specific order. Therefore one gene contains the information of one step of the building sequence. The full building sequence leads to the construction of a complete agent as specified in the genome.

Three operations are encoded in each gene:

1. Rotation of the structure: If this is not the first gene, the already existing structure of the agent can be rotated up to two times, first +/- 90° around the z-axis and second +/- 90° around the y-axis.
2. Preparing a module: A module type is selected and picked from the storage grid. Active modules can be rotated up to two times, again first +/- 90° around the z-axis and second +/- 90° around the y-axis.
3. Connection of the module: The new module is connected to the topmost surface of the already built structure using the HMA. If this is the first gene, the module is placed in the center of the construction workspace.

**Fig 2:**
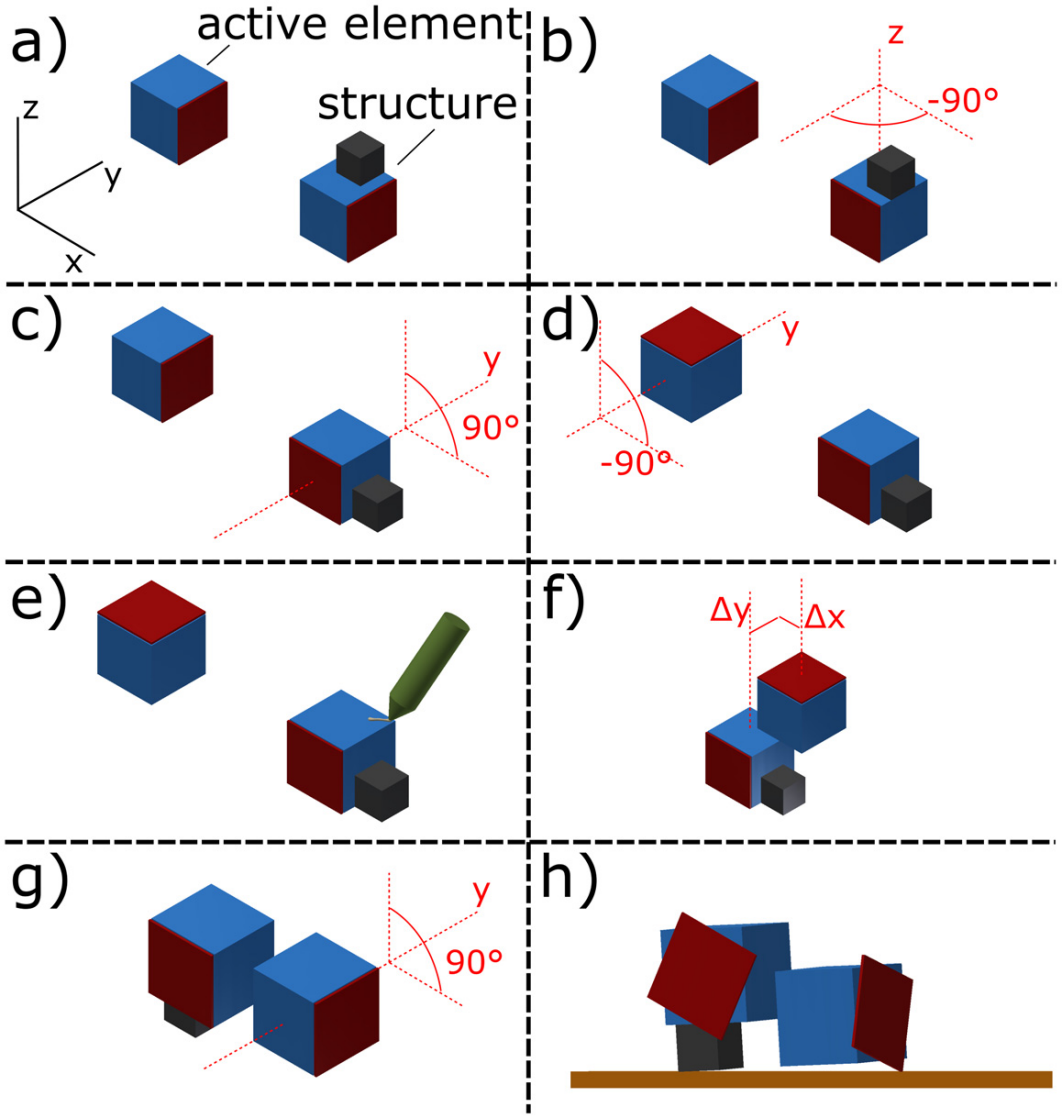
Construction process. This example illustrates the execution of the third (and last) gene of the genome shown in Table 1. (a) The initial configuration shows the state after construction of the first two genes, i.e. a passive element is fixed onto an active module (labeled as structure) and a second active element is prepared. (b-c) Rotation of the structure by -90° around the z-axis and 90° around y. (d) Rotation of the new element by -90° around y. (e-f) HMA is applied to the structure and the module is connected on top of the structure according to the position offset parameters Δx and Δy. (g) An end-rotation of the whole agent by 90° around the y-axis is performed. (h) The motor control parameters are assigned to the active elements and the agent can be evaluated.

With these operations, an evolvable encoding is achieved. The fixed sequence of operations ensures that the endpoint of the operations from one gene can serve as a starting point for the next loop. Therefore, genomes can be adapted, without entering a state that cannot be handled by the system. Table 1 lists a complete example genome consisting of three genes. The construction operations parameterized by the third gene are illustrated in Fig 2. A complete building process is shown in S1 Movie, and for a list of parameter values the reader is referred to the supplementary information in S1 File.

**Table 1:** Example of a complete genome.

Field	Gene 1	Gene 2	Gene 3
structure	[0°, 0°]	[-90°, 0°]	[-90°, 90°]
Type	2 (active)	1 (passive)	2 (active)
orientation	[90°, 0°]	[0°, 0°]	[0°, -90°]
rel pos	[0, 0]	[0, 0.8]	[0.6, 0.8]
Area	0.5	0.8	0.2
phase shift	4	0	3
amplitude	5	0	5
rot end^a^	1		

The construction process associated with the third gene is illustrated in Fig 2, and all parameters are explained in more detail in S1 File.

^a^ Single parameter defined for the whole genome

#### Elite, Mutation and Crossover

After the fitness evaluation of one generation is completed, the agents’ genomes for the following generation are created. The elite of fittest individuals advance unchanged to preserve their abilities. The remaining slots of the next generation are filled with agents whose genomes were altered through mutation or crossover. For each slot, it is randomly determined through which of the two methods the new genome is generated, with a bias towards mutation. In both cases, the probability for an agent to be selected as a parent is proportional to its fitness. All probabilities and parameters are given in S1 File.

The mutation considers three types of modifications to the genome. Either a new gene is added at a random location to the genome; a random gene can be deleted; or a single parameter of a gene is changed at random. Depending on the phenotype, not all mutations are available to enforce sensible agents (e.g. if the agent only consists of one single servo module, the delete gene mutation cannot be selected; see S1 File for a complete list). Except if stated otherwise in the experiments section, all available mutations are equally likely.

For crossover, complete genes from two parent individuals are merged to form a new genome using a one-point crossover scheme. To generate the offspring, the m first genes of the first sampled parent genome are combined with the n last genes of the second parent genome. The inverse combination of the genes is not considered and the unused genes are discarded, therefore only one offspring per crossover is generated to fill a slot in the following generation. Both integers m and n are independently picked at random, and larger or equal to one.

#### Constraints and Validation

In the above construction process with the given hardware implementation, four major building constraints were identified and investigated in detail:

**Mass**: The gripper force can handle agents only up to 550 g, otherwise an agent slips out of the gripper. Therefore, not more than three active modules can be handled.

**Robot arm range**: The construction workspace is limited to 0.3 m in all three directions to avoid infeasible configurations of the manipulator. Therefore the size of an agent is limited to these dimensions.

**Stability**: An agent can only be built if a stable structure results after every operation.

**Add on topmost element**: To avoid collisions of the gripper with the structure, a new element can only be placed onto the topmost element of the structure. This constraint is treated different than the previous because it is a property of the encoding. While the previous constraints are checked for in a validation step, a slightly different connection operation is applied if this constraint is not active. In this case, a module can be added to any top surface of the structure.

Although designed to primarily generate buildable structures, the chosen encoding can produce genomes which cannot be built in real-world, i.e. they violate any of the above constraints. Evolution could come up with creative solutions exploiting such loopholes. In the given setup, this would at the same time lead to more building failures. Therefore, a validation step is introduced, which checks for some constraints every time a new genome is generated, e.g. after mutation. Invalid genomes are removed, and the generation step (e.g. the mutation) repeated. Agents are removed, if they have too few (e.g. no motor) or too many (too heavy) elements; if they are not statically stable; and if the shaft of a servomotor is about to collide with another element upon rotation. The probability that a randomly generated genome fails in the validation depends on its length and the way it is generated. For example if valid genomes with a single gene are mutated, the failure rate is approximately 19%. For genomes with three genes the rate increases to 55%. Both rates were calculated based on random mutations of at least 1000 genomes in each case.

Stability is achieved if the center of mass of an agent lies within its support polygon with a margin of 8 mm to the closest boundary. Since the stability condition has to be fulfilled not only by the final agent but also by all intermediate sub-structures, it is checked after every operation of the building sequence. As soon as one of the operations fails to be stable, the agent is considered as unstable.

The collision check detects statically in the final phenotype, whether the rotating planes of the servo modules are colliding with another module of the agent. A dynamical check simulating the complete motion of the agent is not performed.

#### Fitness evaluation

The fitness of an agent is defined as the distance it travelled from its initial position divided by the testing time. Computer vision is used to automatically measure the distance. The testing time was initially set to 8 s, but reduced to 4 s due to the increasing speed of agents in the last two experiments. Two webcams (Logitech Webcam C930e) record the agent during locomotion, one at an angled view to show the movement and one recording the agent directly from top. The top view is used to run the image analysis, which determines the center of the projected area of an agent before and after the given testing time. A fixed pixel-to-centimeter ratio is applied to convert the extracted shift in the image to the real-world distance. The detailed steps for the evaluation procedure are described in S1 File.

### Diversity Analysis

To measure diversity, a population has to be categorized into groups according to some criteria. In ecology, species diversity is typically used [27]. Here, two different criteria are applied: Behavior and Morphology. Based on these categorizations, two diversities can be calculated.

For the categorization of the agents according to their behavior, the recording of each agent in the testing environment was manually classified based on four features: (1) Discrete interactions with the ground, (2) constant behavior pattern throughout the test, (3) primary rotation of the agent in the horizontal plane and (4) directed locomotion. Four each of these four features, it was decided whether it applies or not. Each unique combination of these features was then considered as a distinct behavior class.

To categorize the agents based on their morphology, a distance measure for the morphologies is needed [28]. Therefore, two quantitative measures of an agent’s morphology are introduced. First, the size of the agent given by the number of its components, and second the agent’s shape factor c = d_tc_/d_ec_, i.e. the ratio between the diagonal of a theoretical cube with the same volume as the agent (d_tc_) and the diagonal of the agent’s enclosing cuboid (d_ec_). A low shape factor is achieved when combining the elements diagonally, and a perfect cube has the largest possible shape factor of one. This quantification method for the morphologies enables to categorize phenotypes according to similar morphological complexity.

The effective number of types is calculated based on the Shannon index to quantify the population diversity, using the above categorizations as different types [27]. The Shannon index is calculated as

$$H\prime =-\sum_{i=1}^{S}p_{i}\text{ln}p_{i},$$

where p_i_ is the ratio of agents which are part of category *i*, and S is the total number of categories [27]. The diversity, measured as the effective number of types then follows as

$$N_{1}=e^{H\prime }.$$

### Experiments

A total of five experiments of artificial evolution were set up, each evolving the agents for the locomotion task. For an experiment, ten generations of ten agents are considered, i.e. 100 agents are evaluated in real-world for each experiment. The parameters for all five experiments are listed below. The first part, experiments 1a-1d are performed with all construction constraints active, while some constraints were relaxed for experiment 2 to assess the potential complexity the approach can achieve. Furthermore, the environment as well as the motor control were adapted for the different experiments. The detailed setup for each experiment is listed below.

#### Experiment 1a

The first generation was initialized randomly with 1 to 3 elements. Some parameters were slightly modified in experiment 1a. The structure and orientation parameters (see S1 File for details) had the size 1x4, allowing a total of four rotations each. The end rotation on the other hand was disabled. Furthermore, for the mutation only the size restrictions were active. This could lead to agents without any actuation. The agents were evaluated on the hard ground. Various sources of noise disturb the evolutionary process. To partially compensate for noise, in this experiment each agent was evaluated five times and the median performance taken as the fitness.

#### Experiment 1b

Rather than recording an agent multiple times, in this experiment each agent was built 3 times, with the servo modules cyclically interchanged. Every version was only recorded once. Again the median of these three measurements was used. A carpet was chosen as a different environment for this experiment to investigate the adaptability of the evolutionary process. The set of possible amplitudes for this and all subsequent experiments was reduced to 10°, 20° and 40°.

#### Experiment 1c

To evaluate purely morphological adaptation, the motor control parameters amplitude and phase shift were fixed in this experiment, and the mutations acting on these parameters disabled. The phase shift was fixed to 90° and the amplitude to 40°. All agents were only built and tested once on the carpet.

#### Experiment 1d

In this experiment, the motor control parameters were reactivated (with the restricted parameter set from experiment 1b), but the ground covered with even softer foam.

#### Experiment 2

To assess the potential complexity that can be achieved, more parameters were changed for this experiment. The initial generation was not randomly initialized, but genomes which performed well in previous experiments were used.

The stability condition and the collision detection were disabled in the validation step, and the motor control parameters fixed again. To promote large morphological changes, the mutation probabilities were adapted. The add/delete mutations have a combined probability of 2/3 to be executed while all the other possible mutations share the remaining 1/3. To further increase the total amount of mutations, the number of the elite is set to 1 such that 9 agents each generation undergo evolution.

To maintain buildability without the stability and collision validation, the building process had to be manually assisted at two points: First, agents can be unstable during the building process, therefore a human operator assisted the building process to prevent unstable agents from falling. The process itself remained unchanged. Second, to prevent the servo modules from damage without collision check, before the evaluation phase all agents were manually inspected and potentially colliding servos disabled for the fitness evaluation. The agents were tested once on the soft foam.

## Results

### Improving Locomotion Performance

**Fig 3:**
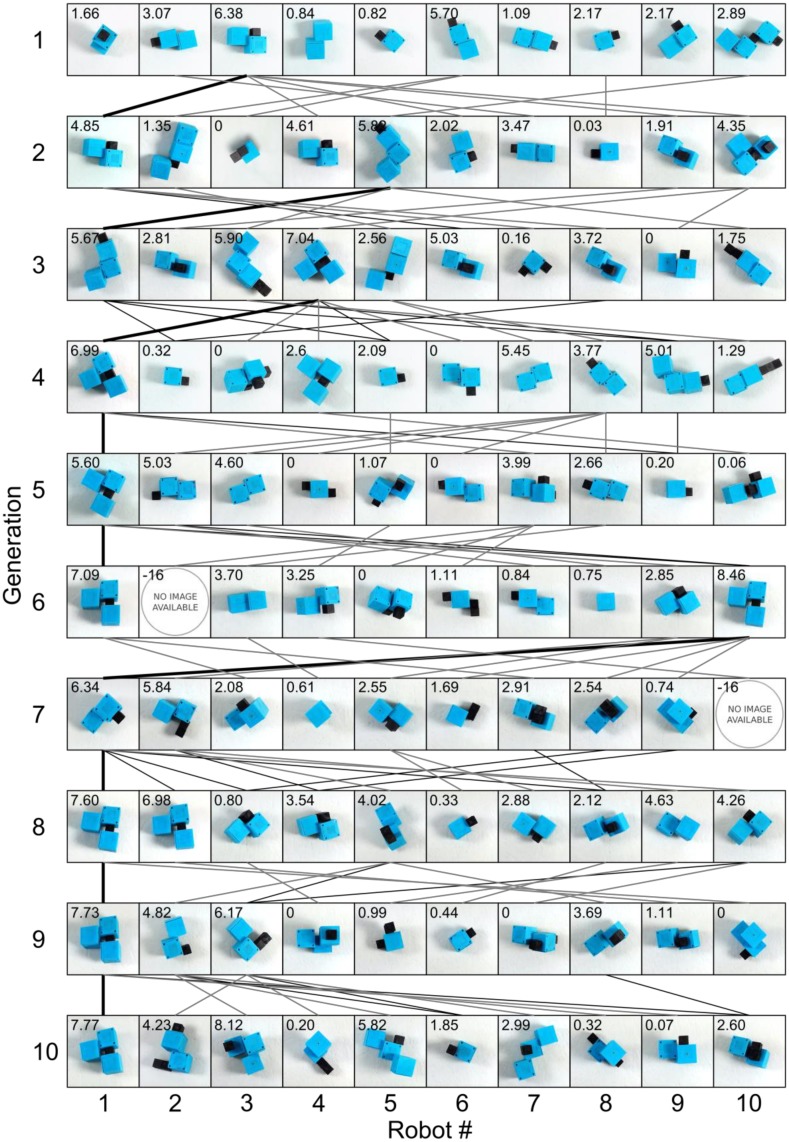
Evolution for a locomotion task. In each of the five experiments, ten generations with ten robots were built and tested. For each robot of experiment 2, an image of its top-view at the beginning of the evaluation process is shown. The number on the top-left corner of each image indicates its fitness (cm/s). The lines between generations show the relations between robots, i.e. the method for generating the new genotype (solid black: elite; thin black: crossover; thin grey: mutation). Negative fitnesses and missing images indicate failure of the building process of the respective robot. The images show that various types of robots are tested, and the fitness of the robots increases in the course of the experiment.

**Fig 4:**
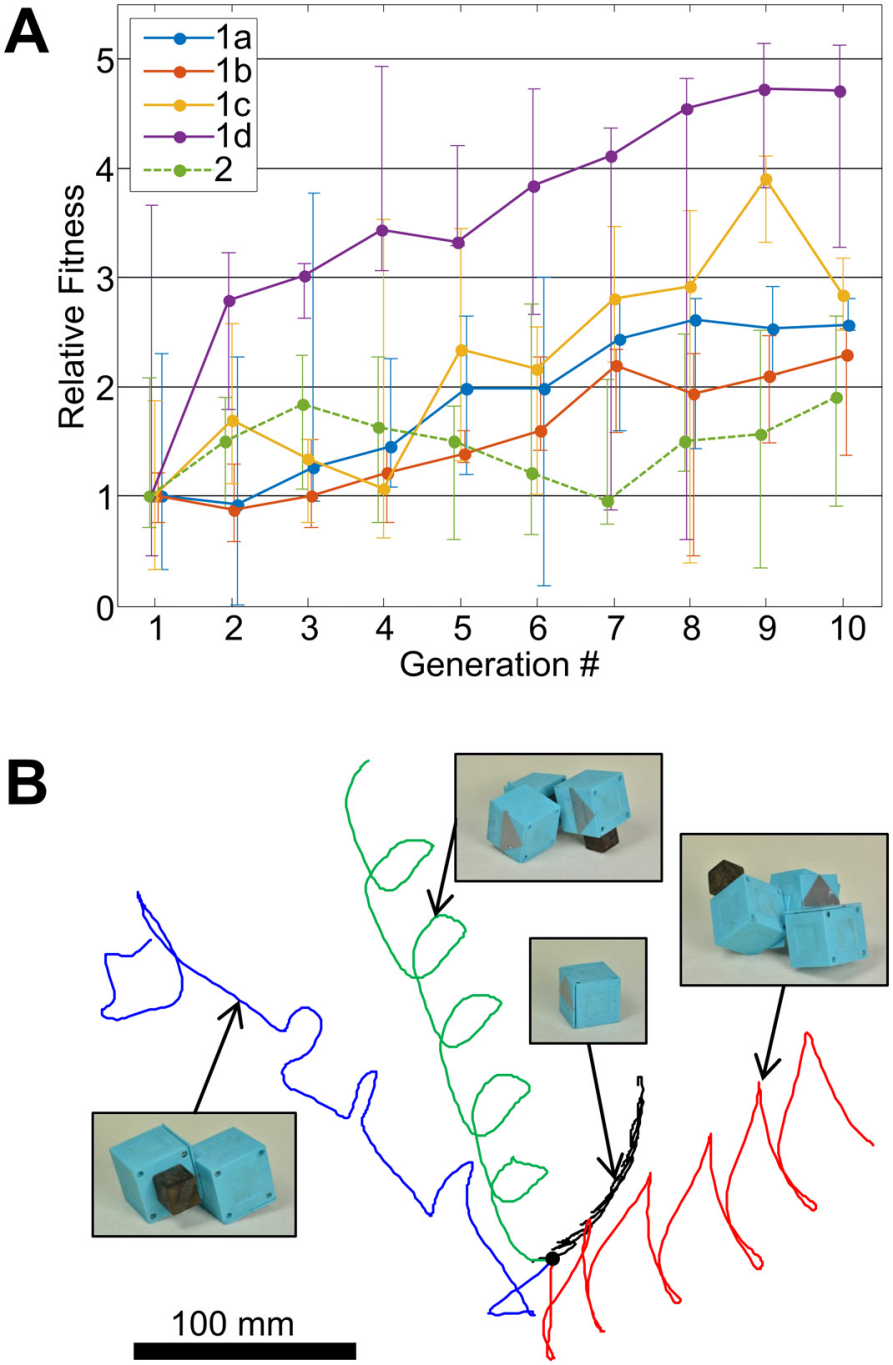
Fitness evaluation and locomotion diversity. In all experiments, the fitness increases relative to the initial generation (A). The lines show the upper quartile of the normalized fitnesses in each generation, with the errorbars indicating best and median fitness. The increasing fitness indicates that the evolutionary process applied to the initial population of robots improves their locomotion capabilities. The top view of the trajectories of four successful robots from experiments 1a, 1c and 2 shows that different locomotion strategies are applied (B). While most successful solutions result in a stable limit cycle, also more unsteady behavior (blue) can achieve a good performance.

The evolutionary process was applied to a population of ten agents over ten generations; i.e. 100 candidates were built and evaluated during an experimental run. An experiment starts with a set of agents, usually randomly generated. Variations of designs are built during evolution and an increase of performance can be observed after a few generations (see S1 Table for the genome data). The increase of fitness was quantitatively analyzed through five such experiments, in which 500 candidate agents were generated by parameter variation, and evaluated in three different environments (Fig 3 and S2 Fig). Except for the first two experiments, each agent was built and evaluated once. In about 96% of all trials, the construction process was successful and the performance of the agent can be analyzed. In the cases where construction fails, zero fitness is awarded to the agent, i.e. it is eliminated in the step to the next generation. In the generation maps, a negative error code is indicating the failure mode (-5: unspecified, -13: HMA connection failure, -14: collision during assembly, -16: other). The normalized fitness of the upper quartile in each generation is plotted in Fig 4A, and a fitness increase of more than 40% over ten generations was observed in all experiments as shown by the results in Table 2. The results in Fig 4A show relatively large fitness variations over generations also for the fittest individuals, although elitism is employed. This is a result of the real-world testing, which includes uncertainties and lacks perfect repeatability. Nevertheless, the overall trends indicate a clear increase of the locomotion fitness during the optimization process.

**Table 2:** Results of the real-world and theoretical experiments.

	Exp. 1a	Exp. 1b	Exp. 1c	Exp. 1d	Exp. 2	Exp. 3
Testing Environment	Real-World	Real-World	Real-World	Real-World	Real-World	Simulation
Ground	Hard	Carpet	Carpet	Soft	Soft	N/A
Active Constraints	1,2,3,4	1,2,3,4	1,2,3,4	1,2,3,4	1,2,4	None
Initial Fitness^a^ (cm/s)	1.6	1.3	3.1	2.8	5.0	N/A
Final Fitness^a^ (cm/s)	2.9	2.9	6.2	6.7	7.2	N/A
Behavioral Diversity	5.2	4.1	8.4	3.2	6.3	N/A
Morphological Diversity	6.1	5.2	7.7	4.0	10.5	N/A
Theoretical Morphological Diversity	8.9	8.9	8.9	8.9	23.2	35.6

^a^Mean fitness of the best three individuals in the first/last generation

In the course of the evolutionary process, many variations of morphologies were generated, eventually resulting in faster, i.e. more fit, individuals (see Table 2). For example, the mean fitness of the best three individuals in the first generation of experiment 1d was 2.8 cm/s, but it increased up to 6.7 cm/s in the last generation. The increase of performance is not only achieved by the fine tuning of design parameters, but also by “inventing” qualitatively different morphologies and gait patterns for locomotion.

Four agents were subject to further analysis of their locomotion patterns: The best agents in the last generation of experiments 1a, 1c and 2, and the second best of experiment 2. Each of these agents were equipped with three reflective markers and tracked with a motion capture system (NaturalPoint, OptiTrack). The trajectory of the midpoint of these markers was recorded while the agent moved on the same ground as in the experiment. From the tracking data, the trajectories in the horizontal plane were extracted and are shown in Fig 4B. While some candidates show stable locomotion patterns, others exhibit rather unsteady trajectories for forward locomotion.

### Diversity and Physical Constraints

To achieve the observed increase of fitness through the course of evolution, a sufficient diversity of solutions is required. The variety of solutions is expressed in the various behaviors the candidate agents exhibit when interacting with the environment in the testing phase. To quantify the behavioral diversity in all five experiments, each agent’s behavior is classified according to four distinct features. Each unique combination of features is considered as a class of behaviors, and based on this classification the behavioral diversity is calculated as introduced in the Diversity Analysis section. The behavioral diversity for all experiments is listed in Table 2.

The categorization of behaviors relies on real-world testing, because the behavior only emerges through the interaction of the robot morphology and its controller with the environment. To enable for a further analysis, a second diversity measure, based on the agents’ morphologies is applied. Each agent is categorized according to its shape factor and size. These parameters can be calculated given an agent’s genome without physically assembling it. The morphological diversity calculated with these measures is also indicated in Table 2. The complete data including behavioral and morphological categorization for all real agents is listed in S1 Table. Behavioral and morphological diversities are two different measures, but both show similar trends. An increased morphological diversity is likely to result in more diverse behaviors, although there is no guarantee.

Many elements in our experiments can influence the emergence of diversity. Four main physical constraints in the automated construction process were identified that dominantly affect the degree of diversity generated in the evolutionary optimization. These are: (1) the payload limit of the mother robot which restricts the maximum weight of a candidate agent, (2) the reachable range of the mother robot that restricts the size of the candidate agent, (3) stability and fixation capabilities of the mother robot while a candidate agent is being built, and (4) the connection capabilities of the mother robot (e.g. whether the mother robot can connect vertical and horizontal surfaces, or only certain configurations).

**Fig 5:**
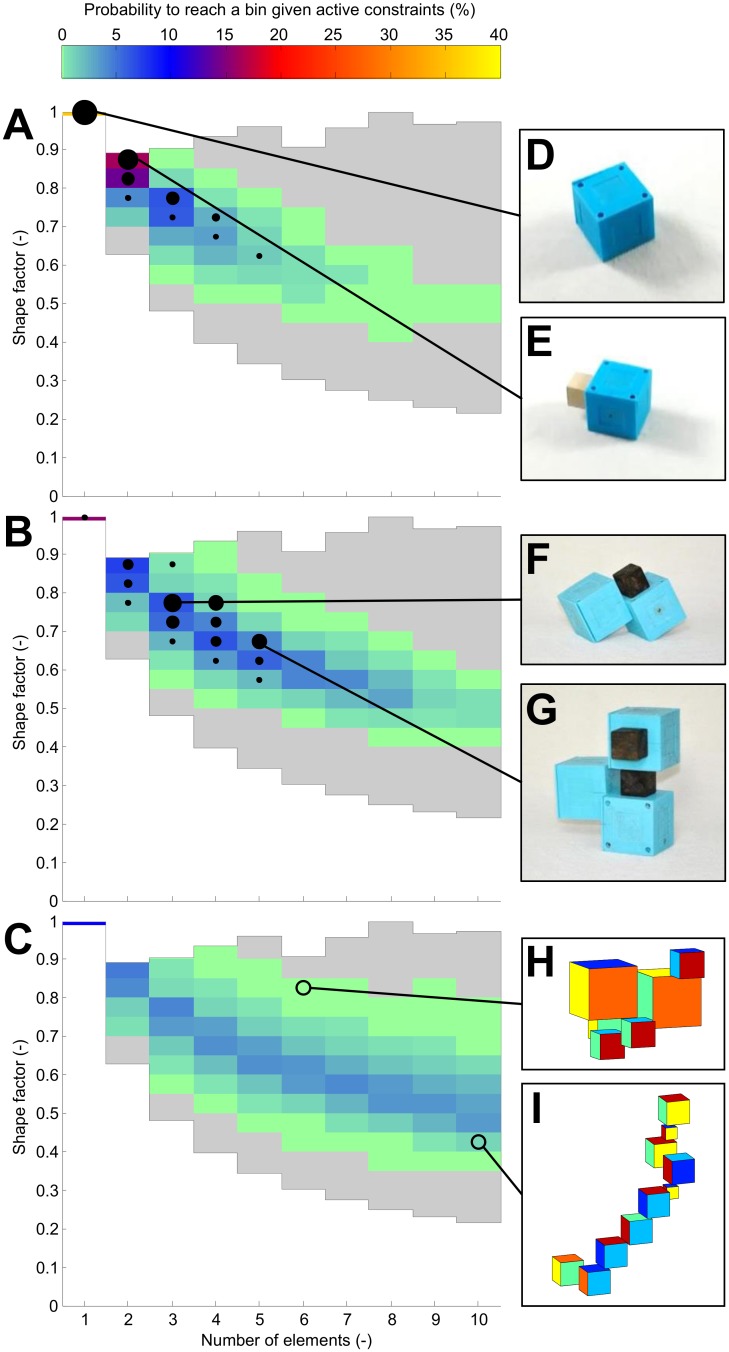
Exploration of the design space. Each agent can be located in the design space given its number of elements and shape factor (A,B,C). The grey area shows the theoretical limit which can be reached using the cubic modules. The portion of this space that can be covered is restricted by the constraints which apply to the real-world construction process. The colored areas illustrate the parts of the design space that can be reached with different sets of active constraints. All constraints are active in (A), the stability condition is relaxed in (B) and no constraints are active in (C). This subsequently increases the reachable portion of the design space. The solid black markers indicate the distribution of the agents in experiment 1b (A) and experiment 2 (B). Their area is proportional to the number of agents in the bin. The right column (D-I) shows two example morphologies per experiment.

To analyze the influence of these four physical constraints onto the diversity that can be achieved, 1.25 million genomes were randomly sampled with uniform parameter distributions and one to ten components. The genomes were then “built” in simulation to calculate the resulting morphologies. For the virtual developmental process, different sets of the abovementioned constraints were activated. First, all constraints were active as in the real experiments 1a-1d, then the stability constraint was removed as in the experiment 2, and eventually all four constraints were relaxed. The resulting morphologies were categorized according to their shape factor and size. From this categorization a theoretical morphological diversity is calculated (Table 2). With this set of randomly generated genomes, categorized based on the corresponding phenotypes and filtered according to the aforementioned four physical construction constraints, the emergence likelihood of each morphology type can be computed. The resulting probability distribution of morphologies is plotted in Fig 5A. In this case, the construction constraints are very strong, thus the distribution is biased towards morphologies with one or two elements and a high shape factor (i.e. the mother robot is less likely to be able to construct complex agents).

Such a restricted trend in evolutionary dynamics can also be observed in the real-world experiments. In the experiments 1a-d (see S2 Fig for the results), with the above physical constraints of the mother robot active throughout the evolutionary process, the populations are dominated by one morphology after several generations. In the experiments 1a and 1b, the resulting morphologies are very simple, and the results in Table 2 suggest that with a final fitness of only 2.9 cm/s, both experiments ended in a local maximum. The morphology distribution of experiment 1b is shown with solid markers in Fig 5A. The following experiments 1c and 1d result in more complex agents, but still their populations are taken over by one type of morphology, with few improvements in the last generations.

In contrast, the breakthroughs of morphological diversity and gait patterns were observed when the physical constraint (3) was relieved in experiment 2. The construction process was set up such that the robotic agent under construction can be manually fixed to ensure postural stability. The effect is shown in Fig 5B, in which the enhancement of morphological diversity can be observed through a wider distribution of morphologies. The real-world experimental run shown in Fig 3 employed this evolutionary configuration, and it can be seen that this experiment does not converge to a single solution, with the optimization still discovering new successful morphologies in the last generation. This experiment resulted in the highest real-world diversity and a final fitness of 7.2 cm/s.

Moreover, an additional theoretical analysis of the physical constraints on the construction process was conducted by also removing the three remaining constraints. Fig 5C shows that the probability distribution of morphologies spreads further out to lower shape factors and a larger number of elements, thus the morphological diversity can be enriched and more complex morphologies of agents theoretically be built. Even though the experimental setup does not allow to conduct such experiments, yet it theoretically demonstrates the scalability of the approach to develop richer morphological diversity with realistic modifications to the hardware setup of the mother robot.

Table 2 shows the computed diversity of all real-world and theoretical experiments introduced earlier. This analysis highlights that the physical construction constraints have a critical impact on the generation of diverse solutions, more than other elements such as environmental conditions. To maintain diverse populations throughout the optimization, which increases the chance to avoid local optima, these constraints must therefore be carefully considered.

## Conclusion

The findings in this article support the importance of systematic investigations of model-free phenotype development and optimization in order to achieve more physically meaningful results from the artificial evolution of machines.

For the successful artificial evolution of physical systems, generating a diverse set of solutions is essential to efficiently explore the design space. This can be challenging, because an open-ended construction process is required, which enables for the fabrication of candidate agents with large morphological variation. In particular the autonomous physical construction of candidate solutions, which was largely underestimated in earlier work, is found to be a key element to generate morphological and behavioral diversity. The careful implementation of the variable construction process allows to explore a large set of solutions with initially unknown morphology. The encoding of the process on the other hand has to consider the construction constraints, while maintaining a large design space. The analysis of physical construction constraints gives rise to a set of design principles for morphological diversification, and the underlying evolutionary dynamics should be applicable to a broad range of engineering design problems.

The demonstrations show the feasibility of the model-free evolution of a physical system. The evaluation of a candidate’s fitness is done with a physical robot, producing real data in a time-intensive process. Simulations on the other hand could test more solutions in shorter time. Therefore, it might be interesting to combine both methods rather than using one extreme with simulation or real-world testing only. Simulation could for example be employed to preselect promising candidates for real-world testing, reducing the amount of time spent on solutions with low or no chance of success.

Extending the model-free phenotype development and optimization could allow machines to autonomously and adaptively modify their mechanical structures together with their control, similar to the animals’ functions observed in ontogenetic developmental processes.

## Supporting Information

S1 Fig Active module. CAD image illustrating the placement of the components (left) and photo of two active modules with shaft in initial position and rotated (right).
(PNG)

S2 Fig Generation maps. Generation maps for all five experiments.
(PDF)

S1 File Supplementary information. Additional information on algorithm details and its implementation, including parameter lists and values.
(PDF)

S1 Movie Building process. The mother robot constructs a locomotion agent from two active and one passive modules. All modules are initially in the storage area on the left side. The robotic manipulator picks the modules from the storage, rotates modules and agent, and uses hot melt adhesive for connection. All parameters for these processes are stored in the agent’s genome. Once the phenotype is built, it is placed in the testing area.
(MP4)

S2 Movie Fitness evaluation. The evaluation of the fitness of the four agents from Fig 4B is shown here. Each agent is placed in the testing area and the active modules are run with the motor control parameters (amplitude and phase shift) specified in the respective genes. The top view was eventually used to extract the distance travelled.
(MP4)

S1 Table Genome data. This data table contains the complete genomes of all agents from experiments 1a-d and 2, as well as the results from the morphology and behavior classification.
(XLSX)
