# Supplementary figures and images for: Morphological Evolution of Physical Robots through Model-Free Phenotype Development
Source: PLoS One. 2015 Jun 19;10(6):e0128444. doi: 10.1371/journal.pone.0128444 (PMC4474803; doi:10.1371/journal.pone.0128444)

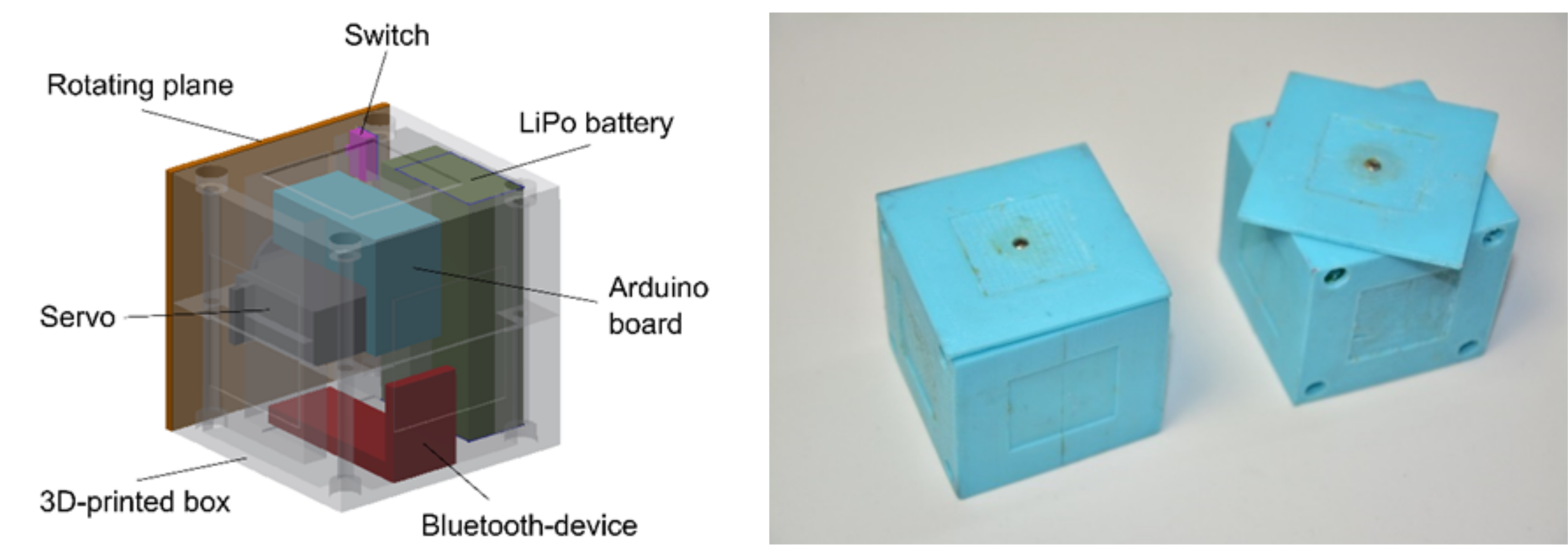

Supplement: S1 Fig — CAD image illustrating the placement of the components (left) and photo of two active modules with shaft in initial position and rotated (right). (PNG) [file pone.0128444.s001.png]
