# Supplementary material for: Morphological Evolution of Physical Robots through Model-Free Phenotype Development
Source: PLoS One. 2015 Jun 19;10(6):e0128444. doi: 10.1371/journal.pone.0128444 (PMC4474803; doi:10.1371/journal.pone.0128444)
Supplement: S2 Fig — Generation maps for all five experiments. (PDF) [file pone.0128444.s002.pdf]

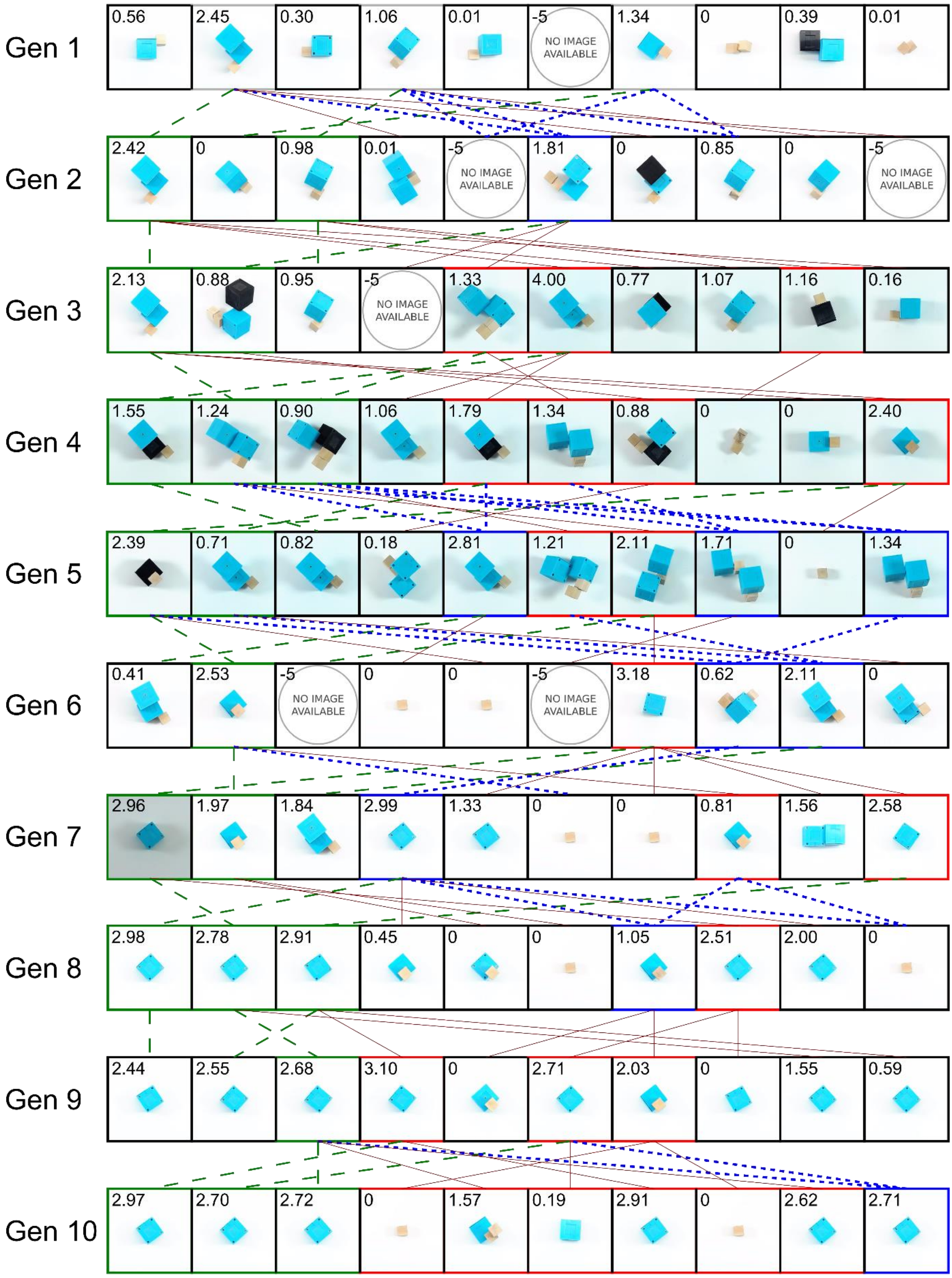

Figure S3.1: Generation map of experiment 1a, evaluated on the hard ground. The evolutionary process, starting from randomly initialized agents, converges to single-servo solution. Green lines indicate individuals selected as part of the elite, red indicates mutations and blue crossover.

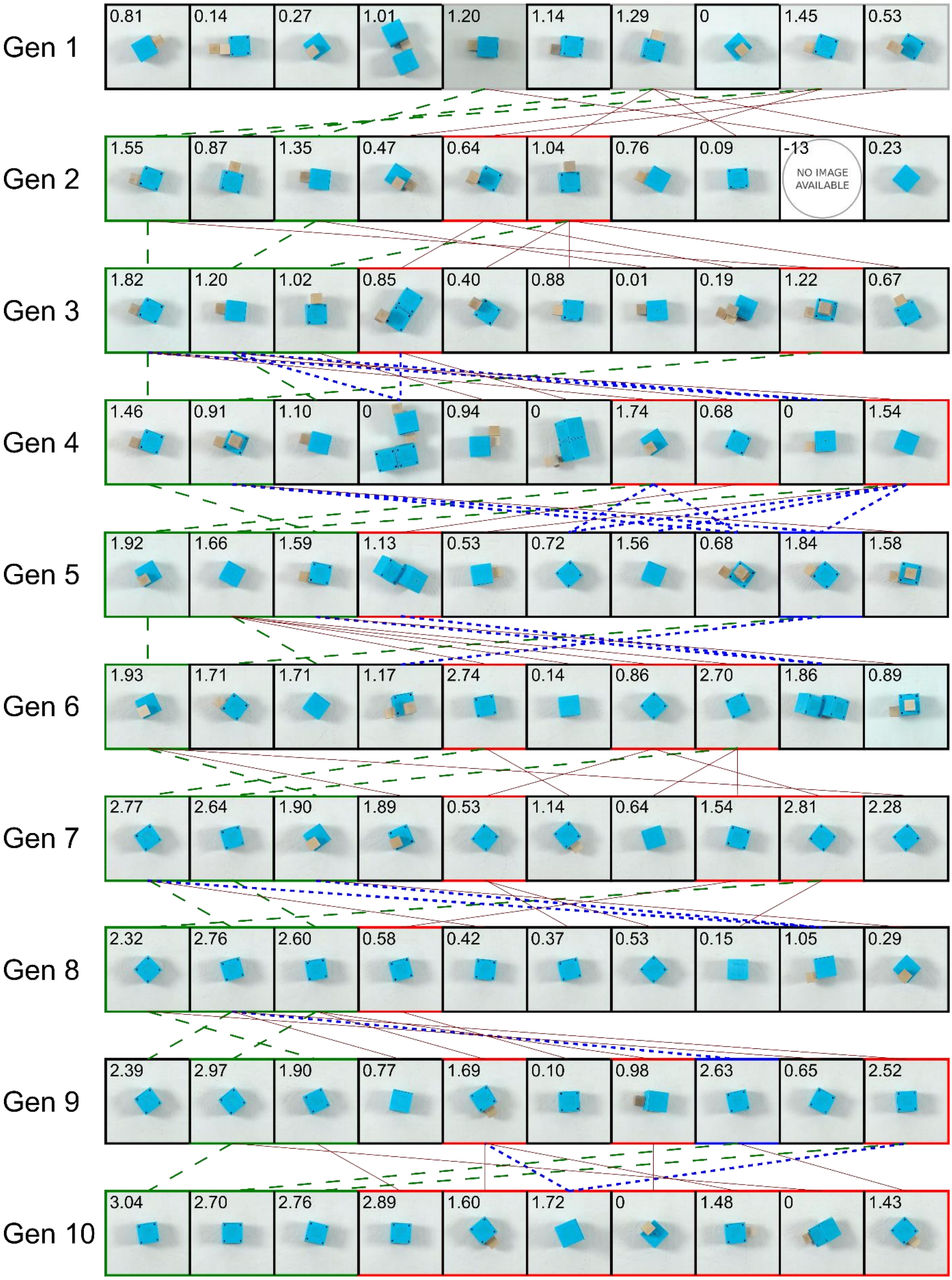

Figure S3.2: Generation map of experiment 1b, evaluated on the carpet. On this different ground, the evolutionary process again converges to a single-servo solution. Green lines indicate individuals selected as part of the elite, red indicates mutations and blue crossover.

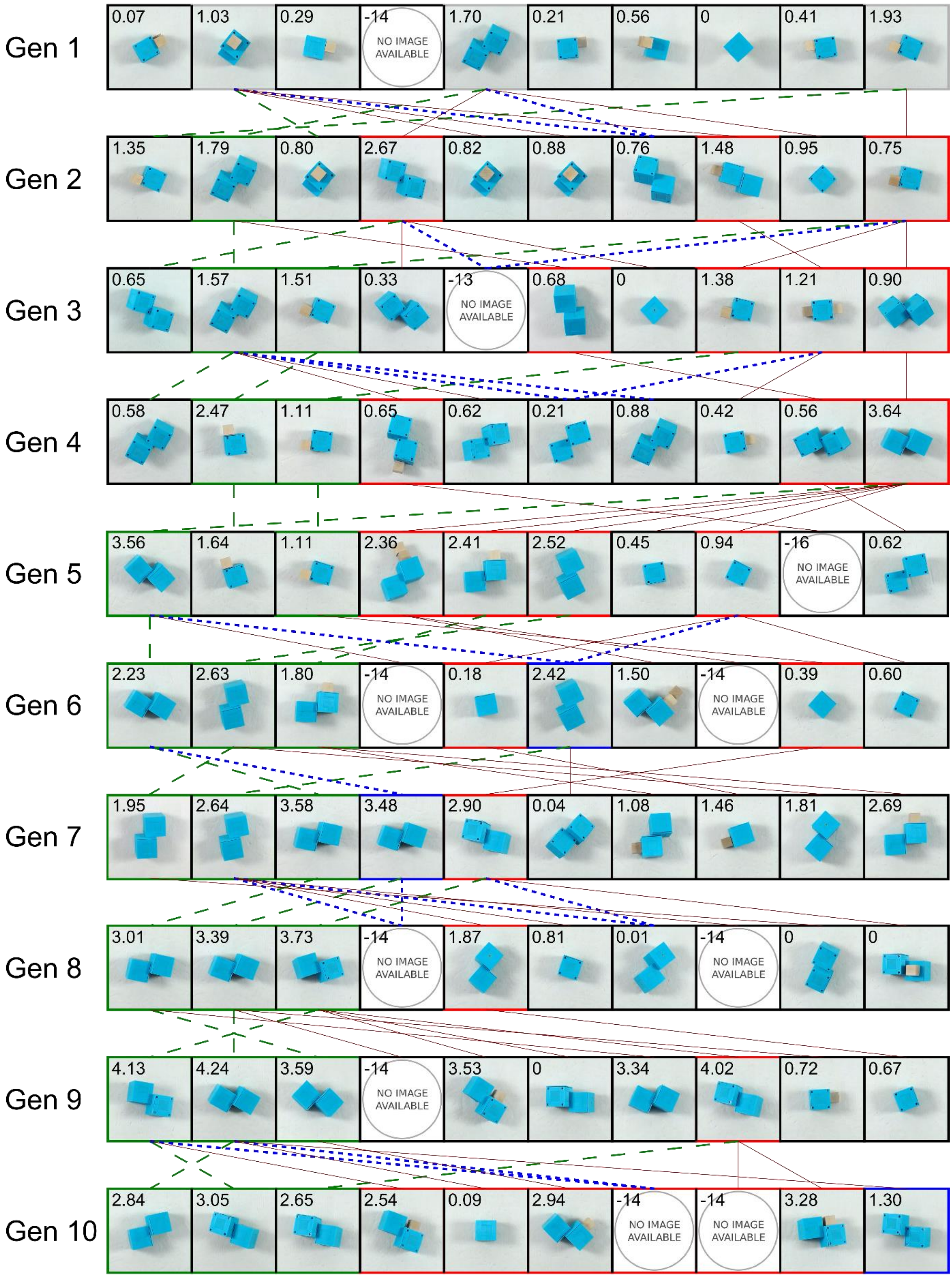

Figure S3.3: Generation map of experiment 1c, evaluated on the carpet with fixed motor control parameters. Disabling the motor control forces the evolutionary process to explore more complex morphologies. Green lines indicate individuals selected as part of the elite, red indicates mutations and blue crossover.

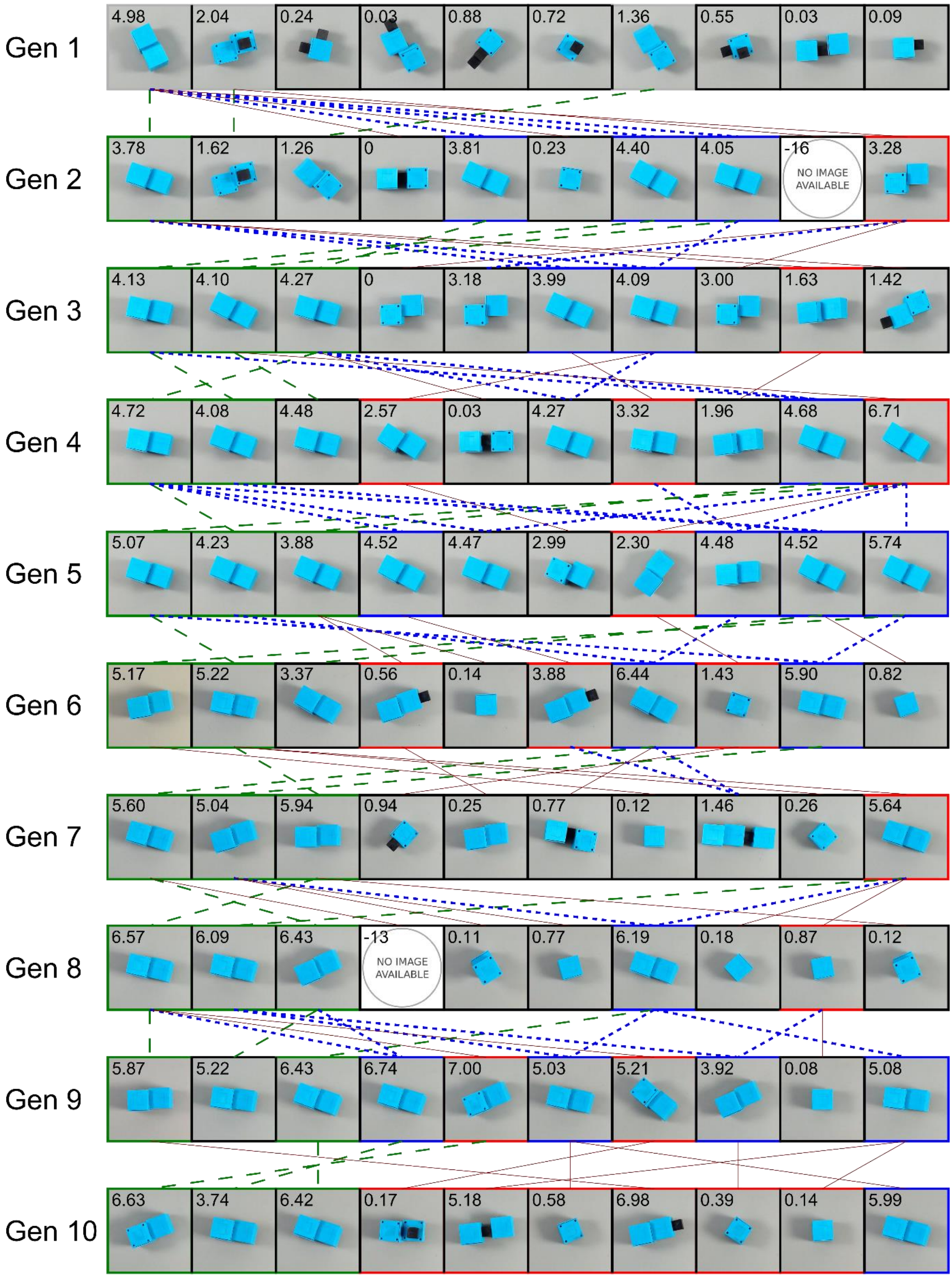

Figure S3.4: Generation map of experiment 1d, evaluated on soft foam. All single-servo solutions perform poorly on this ground. The evolutionary process finds a suitable multi- servo solution. Green lines indicate individuals selected as part of the elite, red indicates mutations and blue crossover.

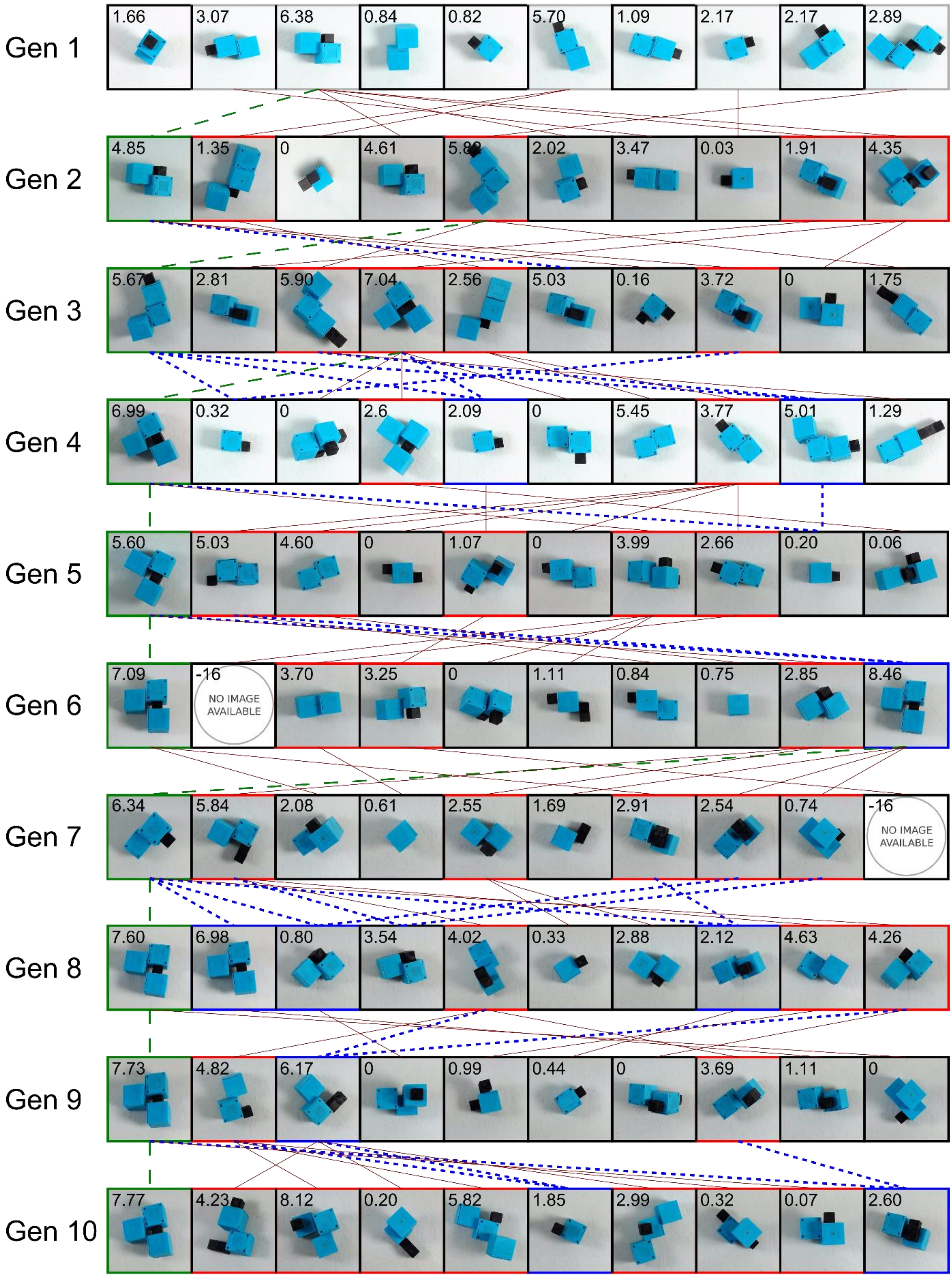

Figure S3.5: Generation map of experiment 2, evaluated on the soft foam. No convergence can be seen in ten generations with the evolutionary process investigating many different multi-servo solutions. Green lines indicate individuals selected as part of the elite, red indicates mutations and blue crossover.
