# Supplementary material for: Morphological Evolution of Physical Robots through Model-Free Phenotype Development
Source: PLoS One. 2015 Jun 19;10(6):e0128444. doi: 10.1371/journal.pone.0128444 (PMC4474803; doi:10.1371/journal.pone.0128444)
Supplement: S1 File — Additional information on algorithm details and its implementation, including parameter lists and values. (PDF) [file pone.0128444.s003.pdf]

S1 File: Supplementary Information

Evolutionary Process Overview

To start the evolutionary process, the first generation of agent genomes is initialized. The "mother robot" builds an agent using its gripper for manipulation and the hot melt adhesive (HMA) supplier to connect the elements. After the assembly, the agent is placed in the testing area where its performance in locomotion, i.e. its fitness, is tested. This process is repeated until all agents of a generation are evaluated. Their performance is recorded with an overhead camera and image analysis is used to extract the distance travelled from the initial position in a given time. Agents are then selected according to their fitness for the next generation. An elite reaches the next generation unchanged, the rest is adapted through crossover and mutation. This process is repeated until a defined number of generations is reached as shown in Figure S1.1.

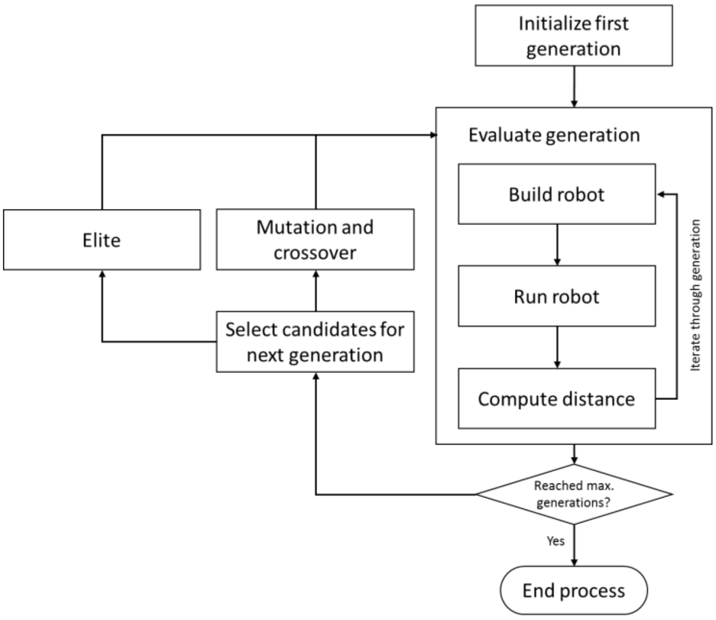

Figure S1.1: Evolutionary process. A first generation is initialized. All agents are built and evaluated, and their fitness is used to select candidates for the next generation. The selected agents can be adapted for the next generation through mutation and crossover.

Algorithm Details & Parameters

Parameters

Table S1.1 summarizes the general parameters for the evolutionary process. These default parameters were applied to all experiments, unless otherwise stated for individual experiments in the respective sections in the main text.

Table S1.1: Parameters of the evolutionary algorithm

Parameter	Value	Description
n_generations	10	Number of generations
pop_size	10	Population size, i.e. number of individuals per generation
n_elite	3	Number of the best individuals transferred to the next generation unchanged
mutation_ratio	0.8	Probability for selected individuals (except elite) to be mutated

max_mutations	3	Maximum number of mutations that can be executed per individual. The number of mutations is a random integer from 1 to max_mutations
crossover_ratio	0.2	Probability of new individuals to be generated through cross-over (crossover_ratio = 1-mutation_ratio)
evolution_tries	20	Maximum number of trials for mutation or crossover. If no valid genome can be created, the original genome is returned
mutation_weights	Uniformly distributed (see Table S1.3)	Probability of each different mutation to be executed

Additionally, the chances for each individual to be selected for evolution are proportional to its fitness.

Assembly Process Encoding

The building sequence encoded in each agent's genome is executed step by step as listed below. An example is illustrated in Fig. 2 and all parameters are summarized in Table S1.2.

1. Rotation of the already existing structure. If no structure exists, ignore this step.
2. Pick the specified module from the storage.
3. If it is an active element, rotate the element. If the element is a wooden cube, ignore this step (wooden cubes do not have a specific orientation).
4. Glue the element to the defined position on the structure. If the element is the first element, place it without gluing. The element now becomes part of the structure.
5. Repeat steps 1-4 for every gene in the genome.
6. If specified, execute one more rotation of the whole agent.
7. Place the agent in the testing area.
8. Assign the motor controls to each servo module and initialize the testing procedure.

Table S1.2: Information in one gene

Field	Size	Admissible Values	Description
structure	1x2	[-90°, 0, 90°]	Rotation of the existing structure before placing a new object. Does not apply to the first element. First column: Rotation around z-axis; Second column: Rotation around y-axis.
type	scalar	1, 2	Type of element. 1: Passive, 2: Active.
orientation	1x2	[-90°, 0, 90°]	Rotation of the element (only applied to servo modules). First column: Rotation around z-axis; Second column: Rotation around y-axis.
rel_pos	1x2	[-1, -0.9, -0.8,… 1]	Defines the relative position of the new element on the topmost surface of the structure. 0: Element is centered; ± 1: Element is placed on the edge of the topmost surface minus a margin of 1 cm for connection. Does not apply to the first element. First column: x-axis; Second column: y-axis.
area	scalar	[0, 1]	If more than one element form the topmost surface, this parameter is used to decide which surface the rel_pos parameters are applied to. Has no influence if only one element forms the topmost surface. Does not apply to the first element.
phase_shift	scalar	[1, 2, 3,… 8]	Defines the phase shift of an active module. Is the index to the possible phase shifts [0°, 45°, 90°, 135°,

			180°, 225°, 270°, 315°]. Does not apply to wooden cubes.
amplitude	scalar	[1, 2, 3, 4, 5]	Defines the amplitude of an active module. Is the index to the possible amplitudes [0°, 10°, 20°, 30°, 40°]. Does not apply to wooden cubes.
rot_end	scalar	0, 1	Defines if the agent is rotated +90° around the y-axis after complete building process before being placed in the testing area. 0: No rotation; 1: Execute the rotation. Only applies to the last gene.

Mutation Probabilities

The following mutations to the parameters specified in a genome are available:

- Add a gene (add)
- Delete a gene (del)
- Change one rotation in the structure parameter (struct)
- Change one rotation in the orientation parameter (ori)
- Change one position in the relative position parameter (rel_pos)
- Change the amplitude of a servo module (amp)
- Change the phase shift of a servo module (ps)
- Change the end-rotation parameter (rot_end)

Depending on the phenotype, not all mutations are available (e.g. if the agent only consists of one single servo module, the delete gene mutation cannot be selected). Table S1.3 shows, which mutations are disabled based on the phenotype. Except if stated otherwise in the experiments section, all available mutations are equally likely.

Table S1.3: Mutation probabilities based on the phenotype characteristics.

**Phenotype**	**add**	**del**	**struct**	**ori**	**rel_pos**	**amp**	**ps**	**rot_end**
no special case	1/8	1/8	1/8	1/8	1/8	1/8	1/8	1/8
single wooden cube	1	0	0	0	0	0	0	0
single servo module	1/5	0	0	0	1/5	1/5	1/5	1/5
only wooden cubes	1/5	1/5	1/5	1/5	0	0	0	1/5
5 elements	0	1/7	1/7	1/7	1/7	1/7	1/7	1/7

Validation Step

Although designed to generate buildable structures, the chosen encoding can produce genomes which cannot be built in real-world. Therefore, a validation step is introduced, which checks for some constraints every time a new genome is generated, e.g. after mutation. Invalid genomes are removed and regenerated. The following types of agents are removed:

- Unstable agents
- Agents where servo-shafts collide with other modules
- Agents with zero elements
- Agents with more than five elements
- Agents with more than three servo modules
- Agents with less than one servo module

The stability condition has to be fulfilled not only by the final agent but also by all intermediate sub-structures. It is therefore checked after every operation of the building sequence. As soon as one of the operations fails to be stable, the agent is recognized as unstable. Stability is achieved if the center of mass of an agent lies within its support polygon with a margin of 8 mm to the closest boundary.

The collision check detects statically in the final phenotype, if the rotating planes of the servo modules are colliding with another module of the agent. A dynamical check simulating the complete motion of the agent is not performed.

Fitness Evaluation Procedure

The fitness of an agent is defined as the distance travelled from the initial position divided by the testing time.

Computer vision was used to automatically measure the distance travelled by the agents. The testing time was initially set to 8 s, but reduced to 4 s due to the increasing speed of agents in the last two experiments. Two webcams (Logitech Webcam C930e) record the agent during locomotion, one at an angled view to show the movement and one recording the agent directly from top. The top view was used to run the image analysis, which determines the center of the projected area of an agent before and after the given testing time. A fixed pixel-to-centimeter ratio is applied to calculate the distance travelled in real-world. The detailed steps for the evaluation procedure are described below:

1. Run the servos for 1s to force the agent into a stable position. Some agents initially fall, producing some displace. This movement shall not be considered in the fitness.
2. Move all servos to the initial position and stop.
3. Activate all servos again for the given testing time and record the movement of the agent.
4. Extract the first and last frame taken and transform them to the Hue, Saturation, Value (HSV) color space. The HSV color space is less prone to noise when detecting a certain color. A beige (in later experiments black) color filter finds the wooden cubes and a blue color filter finds the servo modules, converting the complex images to a binary images as shown in Figure S1.2.
5. Combine the objects found, erode the white patches by a square mask of 3 pixels to get rid of noise, dilute the same patches by a square mask of 7 pixels to combine the single patches to one connected patch and fill in holes (missing pixels of a closed shape) to generate a complete patch of the whole agent.
6. Find the center of area in both frames and calculate the distance travelled with a fixed pixel-to-cm ratio (0.11 cm/pixel).

In Figure S1.2, an example of the image analysis procedure can be seen.

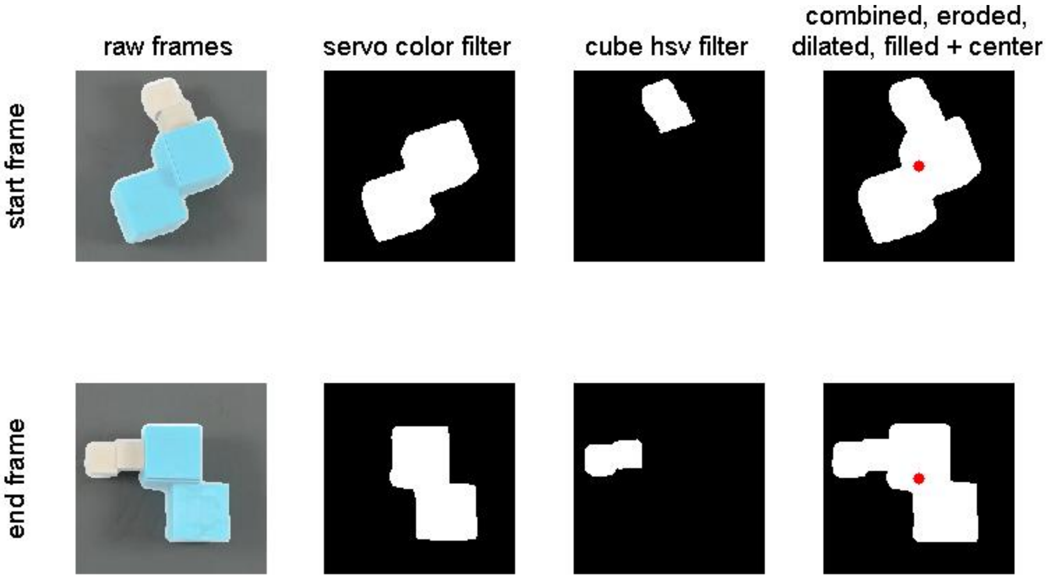

Figure S1.2: Image analysis process. The first column displays the real agent with the patch of the image analysis overlaid after the following three processes: 1. Onto the raw image data, the column titled "servo color filter" applies a blue color filter in the HSV color space to find the servo modules. 2. The next column applies a beige (in later experiments black) color filter to find the wooden cubes. 3. The last column shows the final patch of the agent as the image analysis computes it after combining, eroding, diluting and filling. Indicated in both the start and end frame is the center of area which is used to compute the distance travelled by a pixel-to-cm ratio.

In about 4% of all trials, the construction process failed. In such a case, zero fitness is awarded to the agent, i.e. it is eliminated in the step to the next generations. In the generation maps, a negative error code is indicated (-5: unspecified, -13: glue connection failure, -14: collision, -16: other).

Shape factor bounds

For the modules used in our experiments, a lower and upper bound of the shape factor can be calculated for a given number of elements. With one cubic element, only $c = 1$ can be achieved. With two elements, depending on their configuration, $0.65 < c < 0.9$ can be achieved. As the number of components s increases, the lower bound of c approaches 0, which is achieved with one type of modules connected diagonally. The upper bound fluctuates below or equal to 1. The upper bound was calculated testing all combinations of active and passive modules, e.g. a perfect cube can be obtained with 8 active modules (= 8 elements) or 7 active modules and 8 wooden cubes (= 15 elements). In the plots in Fig. 4, this area is shown in grey.
